# Node-positive breast cancers diagnosed in 2011 at York Teaching Hospitals NHS Trust: an analysis of the adequacy of current preoperative assessment

**DOI:** 10.1186/bcr3311

**Published:** 2012-11-09

**Authors:** A Chandra

**Affiliations:** 1 York Hospital NHS Foundation Trust, York, UK

## Introduction

Preoperative assessment of the axillary nodal status in primary breast cancer using ultrasonagraphy is now established practice and mandated by the NHSBSP. However, the criteria for nodal biopsy are based on the morphological appearance of the imaged nodes. This study's poster proposes that the tumour and grade stage of the primary tumour should also influence the threshold for nodal biopsy with the intention of improving preoperative assessment and thus decreasing morbidity associated with further surgical intervention to the axilla following positive sentinel lymph node biopsy.

## Methods

A retrospective analysis of final nodal status of the 296 surgically treated patients diagnosed with primary breast cancer in 2011 was performed. Data including preoperative assessment of tumour size, grade, axillary node status (preoperative and postoperative) and final outcome was collected. Patients with negative preoperative axillary node status were compared with postoperative node status and the proportion requiring further treatment was ascertained.

## Results

A total of 207 had negative axillary preoperative assessment and underwent sentinel lymph node biopsy (SLNB). Fifty-one had positive sentinel lymph nodes, and of these 9% required further surgery. Sensitivity at the identification of positive axillary nodal disease preoperatively was 0.49 and specificity calculated at 0.99. Preoperative assessment had a positive predictive value of 96% and a negative predictive value of 73%. A positive correlation between axillary nodal involvement and tumour size and grade was observed; that is, 15% in T1G1 tumours to 36% in T3G3 tumours. Patients in the T2G2 group or above were 54% more likely to have a positive SLNB.

## Conclusion

We propose patients staged preoperatively as T2G2 and above should have axillary node biopsy considered despite negative preoperative ultrasound. This may increase the accuracy of axillary preoperative assessment with a subsequent decrease in second operations to the axilla.
